# Reply from Lars Larsson, Nicola Cacciani and Barry Dworkin

**DOI:** 10.1113/JP271014

**Published:** 2015-07-31

**Authors:** Lars Larsson, Nicola Cacciani, Barry Dworkin

**Affiliations:** Karolinska InstitutetStockholm, Sweden

We are grateful for the thoughtful comments regarding our publication in *The Journal Physiology*, as they provide us an opportunity to clarify several points regarding the effects of mechanical ventilation, *per se*, on diaphragm muscle structure and function. In their comments Schellekens and co-workers are surprised by the lack of a significant decrease in diaphragm muscle fibre cross-sectional area (CSA) during the initial 4 days of controlled mechanical ventilation (CMV). Schellekens and co-workers base this on: (1) a documented rapid decline in diaphragm muscle fibre size in response to CMV, (2) lack of assessment of muscle fibre type, and (3) too high an inspiratory fraction of CO_2_ in the inspiratory air inducing a hypercapnia which is suggested to attenuate the CMV induced diaphragm muscle fibre atrophy.

First, in the publications referenced demonstrating a rapid decline in diaphragm CSA in response to CMV, the duration of CMV varied between 18 and 24 h. Further, commercially available rodent ventilators were used in these studies. To our knowledge, the duration of mechanical ventilation using these ventilators are typically shorter than a day or at most for 2–3 days. The major reason for these relatively short durations is that commercially available rodent ventilators cannot maintain life support for longer, adding confounding factors besides mechanical ventilation with a significant impact on diaphragm muscle size and function. This is in sharp contrast to the clinical situation where mechanical ventilation is a lifesaving intervention and frequently used for longer durations than 24 h. The ventilator used in our study allows long-term mechanical ventilation and life support for weeks–months. In the present study, rats were mechanically ventilated for durations varying between 6 h and 14 days (Corpeno *et al*. 2014) and the longest duration a rat has been mechanically ventilated using this ventilator is 96 days (Dworkin & Dworkin, 1990). In a previous study from our group in mechanically ventilated pigs, where the same type of ventilator was used as in hospital ICUs (allowing long-term life support), diaphragm muscle fibre size was not affected by 5 days CMV, irrespective of muscle fibre myosin heavy chain (MyHC) isoform expression, but a dramatic loss in muscle fibre specific force was observed in all muscle fibre types (Ochala *et al*. 2011). Others, on the other hand, have reported a compensatory increase in diaphragm muscle fibre size in response to 5 days mechanical ventilation in pigs (Radell *et al*. 2002). A small, albeit not statistically significant, decline in diaphragm muscle fibre size was observed in the Sprague–Dawley rats in our study. However, in a more recent study on the effects of CMV on diaphragm muscle fibre structure and function in F344 BN hybrid rats from our group, a compensatory hypertrophy was observed after 5 days CMV in both young and old animals, together with a significant decline in specific force (Cacciani *et al*. 2014). Thus, there may be strain and species differences in diaphragm muscle fibre size in response to CMV. However, when ventilators are used which allow long-duration life-support, then there is no dramatic decline in diaphragm muscle fibre size within 4 days CMV but a significant decline in force generating capacity (specific force).

Second, the MyHC isoform expression was determined on sensitive silver-stained 6% SDS–PAGE in diaphragm muscle cross-sections and in all diaphragm muscle fibres included in the contractile measurements in this study. During the 14-day mechanical ventilation there was no significant change in overall diaphragm MyHC isoform expression or a systematic bias in the selection of muscle fibres expressing specific MyHCs in the time-resolved analyses ranging from 6 h to 14 days. Thus, fibre type differences do not underlie the lack of a dramatic decrease in diaphragm muscle fibres during the initial days of CMV.

Third, the authors´ hypercapnia data cited are interesting but the premise of the comment, as it relates to our preparation is not correct. Although the 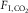 in our preparation is ∼0.03, the alveolar concentration of CO_2_ is probably close to normal, and, more important, the expiratory CO_2_, which is monitored continuously, breath by breath, during the experiment is 37–44 mmHg. The explanation for this is as follows: The tracheal cannula in our experiments is a special coaxial design, with inspiratory and expiratory channels that remain separate to the tip, which is located close to the carina. This has two consequences: (1) The flow in the expiratory channel is unidirectional, so that bronchial secretions are continuously removed, preventing obstruction and allowing very long-term ventilation. (2) Dead space, and thus rebreathing, is minimized; hence, to maintain physiological alveolar concentrations, we add approximately 3% CO_2_ to the inspiratory gas mixture. We no longer routinely measure 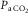, but the preparation has been in use for more than 40 years, and there is a large body of data verifying the correlation of blood gas with expired gas measures. In sum, with the ventilator and tracheal cannula employed in our study, 0.03 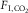 emulates the alveolar gas concentrations of normal ventilation, and does not produce hypercapnia.

Finally, our results using a novel experimental intensive care unit model where rats are exposed to CMV and immobilization, but without confounding factors related to underlying disease or a dying animal, demonstrate a dramatic loss in diaphragm muscle fibre force generating capacity which precedes a statistically significant diaphragm muscle fibre atrophy. The experimental model offers an unprecedented opportunity to study the mechanisms underlying the ventilator-induced diaphragm muscle dysfunction observed in mechanically ventilated intensive care unit patients and the introduction and evaluation of novel pharmacological intervention strategies which can be translated to clinical research and treatment.
